# The impact of digital transformation and earnings management on ESG performance: evidence from Chinese listed enterprises

**DOI:** 10.1038/s41598-023-48636-x

**Published:** 2024-01-08

**Authors:** Lang Wang, Sheng Hou

**Affiliations:** School of Finance, Dongbei University of Finance and Economics, Dalian, Liaoning China

**Keywords:** Environmental social sciences, Energy science and technology

## Abstract

The improvement of enterprise ESG performance is one of the key driving forces to achieve the goal of economic and social green development. There is a gap between knowledge and practice in the ESG performance of Chinese enterprises, and digital transformation (DT) provides new ideas for ESG development. The research purpose of this paper is to explore the impact mechanism of DT on ESG and the specific path of DT to drive ESG. It provides a reference for listed enterprises to rely on DT to empower their sustainable development capability. This paper takes the panel data of A-share listed enterprises from 2011 to 2021 as the sample and measures the core indicators using the text mining method, modified Jones model, and Roy-Chowdhury model. On this basis, using a combination of econometric models and qualitative comparative analysis, we empirically analyze the impact mechanisms of DT on ESG as well as the specific grouping paths that drive ESG performance. The main conclusions are shown as follows. First, DT can significantly reduce ESG, with an impact coefficient of − 0.013, which is significant at the 5% level. It reflects that the DT of enterprises at this stage has certain deficiencies. There is a matching lag in the enterprise's internal organizational resources. The entry of digital technology will have a certain impact on traditional operations, and the high uncertainty of DT adds some hidden costs to the enterprise. Secondly, there is an indirect suppression effect of accrued earnings management (AEM) in the transmission mechanism of DT affecting ESG. It is verified that DT can reduce information asymmetry and inhibit EM activities, thus reducing the impact on ESG. Finally, a total of six configurations achieved high ESG valuations. High technology practice-low performance manipulation; digital intelligence-low performance manipulation; digital intelligence-digital resources; digital resources-inadequate digital infrastructure.; high technology practice-bottom technology deficiency; digital intelligence-high performance manipulation. Through configuration analysis, the suppression effect of EM is further verified. The lack of AEM is usually the core condition of the high-valuation group. Meanwhile, digital intelligence, digital resources, and digital technology practice can drive the improvement of enterprise ESG. The instrumental variables approach and robustness tests support these findings.

## Introduction

The rapid development of the economy has brought about serious environmental problems. It is the focus of society to improve the ability of sustainable development while maintaining the stable operation of the economy^1^. As the micro-main body of economic operation, the improvement of the sustainable development ability of enterprises is one of the key driving forces to achieve green development goals^2^. ESG (Environment, Social, Governance) performance is a comprehensive consideration of corporate environmental responsibility, social responsibility, and internal governance. Compared with traditional financial performance, ESG performance can better highlight the long-term development ability of enterprises. It is a key indicator to conform to the concept of green development and measure the sustainable development ability of enterprises^3^. However, in the past, the long-term extensive development mode has reduced the ESG performance of enterprises. According to the “China ESG Development Report 2021” released by the China ESG Research Institute, the proportion of A-share listed companies that make pure ESG performance disclosure is only 1.57%. At the same time, the ESG Research Report of China's Listed Companies (2021) shows that the overall score of ESG evaluation of China's listed companies is low, and only 8.07% of enterprises are rated as A-level. Therefore, there is still huge room for improvement in the ESG performance of enterprises.

With the rise of the digital economy as a national strategy in China, a new perspective for enterprise ESG development has been opened up (*Made in China 2025*; *the 14th Five-Year Plan for National Economic and Social Development and the Outline of 2035 Vision Goals*; *the 14th Five-Year Plan for the Development of Intelligent Manufacturing*). Digital technology and innovation have brought opportunities but also disrupted traditional industries. This encourages traditional enterprises to carry out digital transformation (DT), driving management organization change and business model innovation^4^. Currently, the DT of enterprises is becoming an important factor affecting the sustainability of enterprises. On the one hand, DT can optimize production operations, improve organizational efficiency, and enhance innovation capabilities. Thus, it can realize the business transformation of enterprises and enhance their profitability and sustainability^5,6^. However, the process of integrating digital technologies into the real economy may have some impact on traditional organizational systems. When an organization's management systems and capabilities do not match or lag behind the technological architecture used for digital transformation, the benefits of digital transformation may be offset by the management costs derived from it. It is difficult to generate benefits^7,8^. On the other hand, DT can transform a vast amount of operational data into digital resources and significantly improve the information transparency of the company. Thus, it improves managers' earnings manipulation behavior and indirectly affects ESG performance^9^. In summary, it is of great significance to analyze the direct and indirect impacts of DT on the ESG performance of enterprises and how to promote the ESG performance for digital technology to enable green development and promote the process of circular economy.

## Literature review

Along with the improvement of the ESG evaluation system, ESG performance has gradually become an important reflection of companies' achievement of social benefits^10^. In the United States, institutional shareholders are under pressure from customer demand and capital flow to continuously improve ESG performance^11^. A large literature explores the factors that influence ESG performance in terms of internal characteristics and external conditions of firms. External characteristics focus on analyzing the impact of external stakeholders such as government, creditors, media, and consumers on ESG performance. For example, after a change of mayor, firms increase their social donations to maintain their relationship with the new government chief executive^12^. Banks are more likely to lend cash to companies with good ESG characteristics to support ESG behavior^13^. The media can influence public perceptions of the ESG performance of listed companies, so companies will meet public expectations by promoting ESG performance^14^. Socially responsible companies can enhance the level of supplier responsibility^15^. Internal characteristics focus on managerial characteristics, such as personality traits like family status or experience, and investors' investment decisions. Personality characteristics such as managers' experience and family status can affect corporate ESG performance. Female board members will be more concerned about social and environmental issues and companies will have higher ESG performance^16,17^. Institutional investors' socially responsible beliefs have a significant impact on socially responsible investment^18,19^. Dyck et al. have found that companies with better ESG performance tend to have investors with longer investment horizons. The analysis of digitization and ESG is still in its infancy^18^.

DT, as a necessary path for enterprises to upgrade their business models and long-term development, has become a hot topic of research for many scholars. Currently, the analysis of the impact of digitalization on enterprises mostly focuses on financial performance^6,20–22^. Some scholars have paid attention to the positive impact of DT on non-financial performance such as social responsibility. Kwilinski et al. tested the spatial spillover effect of digitalization on ESG performance in EU countries from 2008 to 2020. The empirical results show that the European Union has different degrees of ESG performance, DT has the potential to improve ESG performance, and shows a significant spatial spillover effect^23^. Fang et al. investigated the direct impact of corporate digitalization on ESG performance using Chinese-listed companies from 2012 to 2020. It has been found that the digitization of firms significantly improves governance (G) scores and social (S) scores through two channels: reducing agency costs and improving corporate goodwill^24^. The effect on the environmental score (E) is not significant. Ren et al.^25^ have found that digital finance can influence corporate ESG performance through green innovation and external regulation. Su et al.^26^ take Chinese A-share listed companies from 2011 to 2020 as samples and believe that DT has a positive impact on ESG performance, while dynamic capability plays a positive intermediary role. Wu and Li^27^ also adopted a multiple linear regression model to discuss the positive impact of DT on ESG performance and affirm the positive effects of green innovation. Zhong et al.^28^ analyzed the internal mechanism of DT on ESG from the perspectives of curbing management's short-sightedness; improving the transparency of internal information; and improving the technological innovation ability. Lu et al.^29^ proposed that DT can enhance ESG performance by strengthening internal control and green innovation. In the early stage of DT, enterprises will have certain impacts and fluctuations on their original operations. At the same time, DT, especially the application of big data and blockchain technology, can significantly improve the transparency of enterprise information systems. It is conducive to enhancing the efficiency of internal information communication and improving the supervision mechanism, thus increasing the cost and difficulty of earnings management^5^. Current studies focus on the positive effects of DT and the lack of attention to shock fluctuations and suppression variables in the transition process. In addition, most of the existing literature adopts a single quantitative analysis, focusing on the single quantitative relationship. There is a lack of specific path identification and summary of the complex causes that promote ESG performance.

The possible innovations of this paper are as follows: First, in terms of mechanism, the earnings management variable is introduced as a suppression variable to analyze the direct and indirect effects of the impact of DT on ESG. Second, in terms of methodology, the quantitative analysis is combined with qualitative analysis. Based on exploring the overall relationship, this paper puts forward the specific path for listed enterprises to drive ESG performance improvement. There is a causal complexity in the DT to promote the improvement of ESG performance. This paper considers the impact path of multiple conditional elements such as digital transformation internal and EM that overlap and work together to drive ESG. Compared to previous studies that focus on simple linear symmetry and the significance of individual variables, the combination of quantitative and qualitative methods is more likely to provide specific path guidance for listed enterprises to promote ESG performance. Third, in terms of sample selection, the data of Chinese A-share listed enterprises from 2011 to 2021 are used for the first time to analyze the impact effect of DT from both theoretical and empirical aspects.

## Research hypothesis

**Figure 1:**
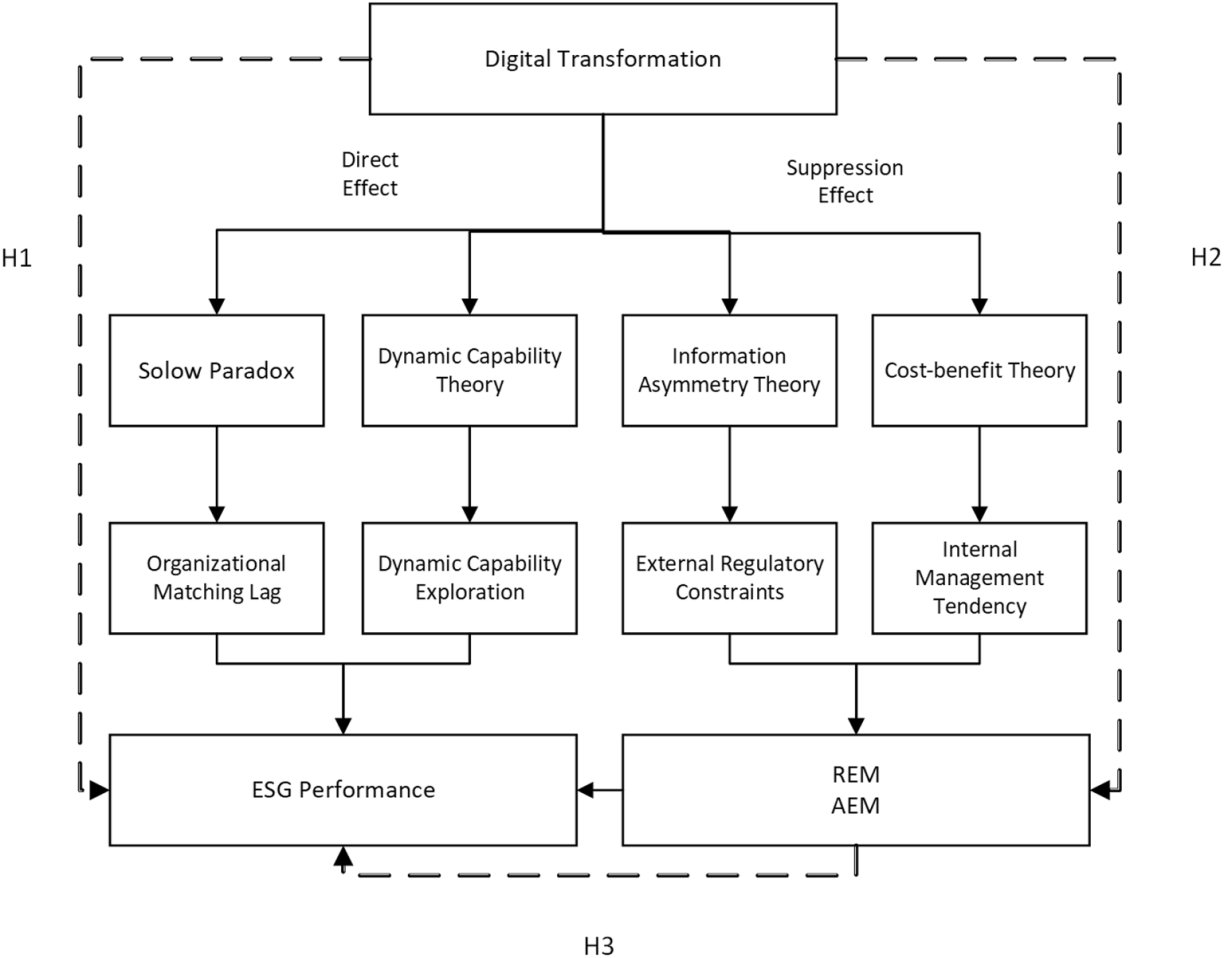
The transmission mechanism.

To further analyze the effect of DT on corporate ESG performance, based on existing theoretical and empirical studies, this paper draws Fig. 1 to describe the impact mechanism and proposes the following three hypotheses.

Brynjolfsson et al.^30^ proposed the productivity paradox by summarizing the phenomenon of the "Solow Paradox". In the context of the digital economy, when digital technologies are integrated into the production chain of a company, digital technologies change the traditional internal operational processes. It requires the coordination of multiple processes such as production and operation, sales, and management, increasing the pressure on profitability. In the short term, it triggers a holistic disorder and reduces the efficiency of business operations^31^. Meanwhile, according to the IT stage theory proposed by Nolan in 1976, enterprise informatization has experienced a complex development process from low level to high level. The journey of organizational change requires increased management costs to obtain a return on IT investment. DT is a long-term process. There is a time lag in matching the management organization status and system with the digital technology architecture of the enterprise. It is necessary to carry out digital transformation step by step according to the development situation of enterprises, industry conditions, and future planning. At the same time, digital change means high uncertainty and increases hidden costs for enterprises^32,33^. The introduction and proficient application of digital technologies also require increased management costs and significant innovation investments. Driven by self-interested behavior, companies tend to defend the interests of their owners when facing the impact of DT. They tend to neglect their environmental and social responsibilities and ignore the interests of other stakeholders' needs. That is, the volatility brought by DT has an impact on ESG performance, and DT can significantly reduce corporate ESG performance.

In summary, hypothesis 1 is proposed: DT has a negative impact on corporate ESG performance.

AEM (Accrued Earnings Management) is the use of accounting standards, through accounting policies to adjust accounting data, and then manipulate earnings. This practice is highly susceptible to the reversal of manipulated accrued profits in a short period, thus putting the firm in financing difficulties and causing a decline in corporate performance^34,35^. REM (Real Earnings Management) is mainly carried out through other means such as production manipulation, stock reversal, sale of assets, expense manipulation, and sales manipulation. These manipulation methods tend to increase the risk of business operations, build up inventory, and cause inflexible capital flows. From the perspective of the stakeholder value reciprocity effect and the insurance mechanism of corporate social responsibility^36^, business operators tend to use accounting methods for accrued earnings management from a utilitarian perspective. It weakens the reliability and predictability of accounting earnings information. This leads to a decrease in the trust of stakeholders and damages the corporate image, thus reducing the ESG performance^37^. The expense manipulation in REM, which contains actions such as compression of innovation investment and reduction of employee overhead, can affect the level of corporate environmental responsibility, social responsibility, and internal governance. At the same time, companies with intensive EM (Earnings Management) have relatively low internal governance mechanisms and ethical constraints of management. The fulfillment of external non-performance responsibilities such as corporate social responsibility and environmental responsibility is lower, and thus ESG performance is more likely to be neglected.

In summary, hypothesis 2 is proposed: There is a negative impact of earnings management on corporate ESG performance.

The DT of enterprises, especially the application of big data and blockchain technologies, makes the activities of enterprises recordable and traceable. It improves the transparency of information within the enterprise and reduces the information asymmetry between stakeholders and the enterprise^38^. Internally, DT can improve the quality of value creation and thus reduce the propensity of management to engage in EM. In terms of external regulation, DT reduces the cost of regulation and increases the constraints on management's business practices. It reduces the scope for EM and improves corporate governance^39,40^. At the same time, the application of digital technology can also improve ESG information quality and management capabilities, and reduce the cost of ESG information management and disclosure^41^, thus reducing the adverse effects of EM on ESG performance. To sum up, DT can restrain the EM behavior of management, thus reducing the impact of DT on ESG performance. By suppression variables, the negative effects of DT on ESG performance are reduced, that is, EM plays a suppression effect in the impact of DT on ESG.

In summary, hypothesis 3 is proposed: DT can reduce the impact on ESG performance by inhibiting EM, that is, EM has a suppression effect.

## Data and research methods

This paper uses the text mining method, modified Jones model, and Roy-Chowdhury model to measure the core indicators. On this basis, the econometric model is used to analyze the effect of DT on ESG performance, and the qualitative comparative analysis is used to explore the specific path of DT driving ESG performance.

### Data

In this study, the data of Shanghai and Shenzhen A-share listed companies from 2011 to 2021 are selected as the initial sample, and ST enterprises, financial enterprises, and samples with missing data are excluded. Meanwhile, continuous variables at the micro level are log-transformed, and on this basis, 1% and 99% tail-shrinking are performed to maintain smoothness and reduce the effect of outliers. We use the raw data provided by the China Stock Market Accounting Research (CSMAR), as well as the relevant corporate annual report data provided by the official websites of the Shenzhen Stock Exchange and Shanghai Stock Exchange to ensure the compliance and scientific validity of the study.

### Core variables

#### Dependent variable

The deepening of the "double carbon" strategy makes the significance of a green economy and sustainable development more and more important. Rapid economic development consumes a lot of resources, leading to a decrease in resources and energy shortage. ESG is an integrated expression of environmental, social, and corporate governance, a value concept, investment strategy, and evaluation tool. It is an important indicator for assessing corporate sustainability^42^. Huazheng’s ESG evaluation system uses AI (Artificial Intelligence) technology to build a big data engine. The evaluation indicators include company news media data, government data, social responsibility reports, and public disclosure data. The system refers to the international mainstream ESG evaluation system and combines the characteristics of the Chinese market. The system has the environment, social, and corporate governance as its three pillars, including 14 themes, 26 key indicators, and more than 130 sub-indicators. Environmental indicators cover aspects such as environmental management systems, green management goals, and green products. Social indicators include the social responsibility system, business activities, social contribution, and other aspects. Governance indicators include aspects such as governance structure, operational risks, and external disciplinary actions. It is updated frequently (quarterly), covers all A-share listed companies, and has high data availability. To consider industry characteristics, the indicator system refers to Thomson Reuters' materiality matrix to construct the industry weighting matrix. It is also based on the systematic measurement of ESG scores of all A-share listed companies over the past 10 years. The total score is 100, and accordingly, nine ratings of C, CC, CCC, B, BB, BBB, A, AA, and AAA are given, with over 20,000,000 ESG evaluation data. Therefore, referring to Fang et al.^24^, and Deng et al.^43^, a score of 9–1 is assigned based on the quarterly ESG ratings of Huazheng, and the annual average is taken to represent the ESG performance of companies.

#### Core explanatory variable

**Figure 2:**
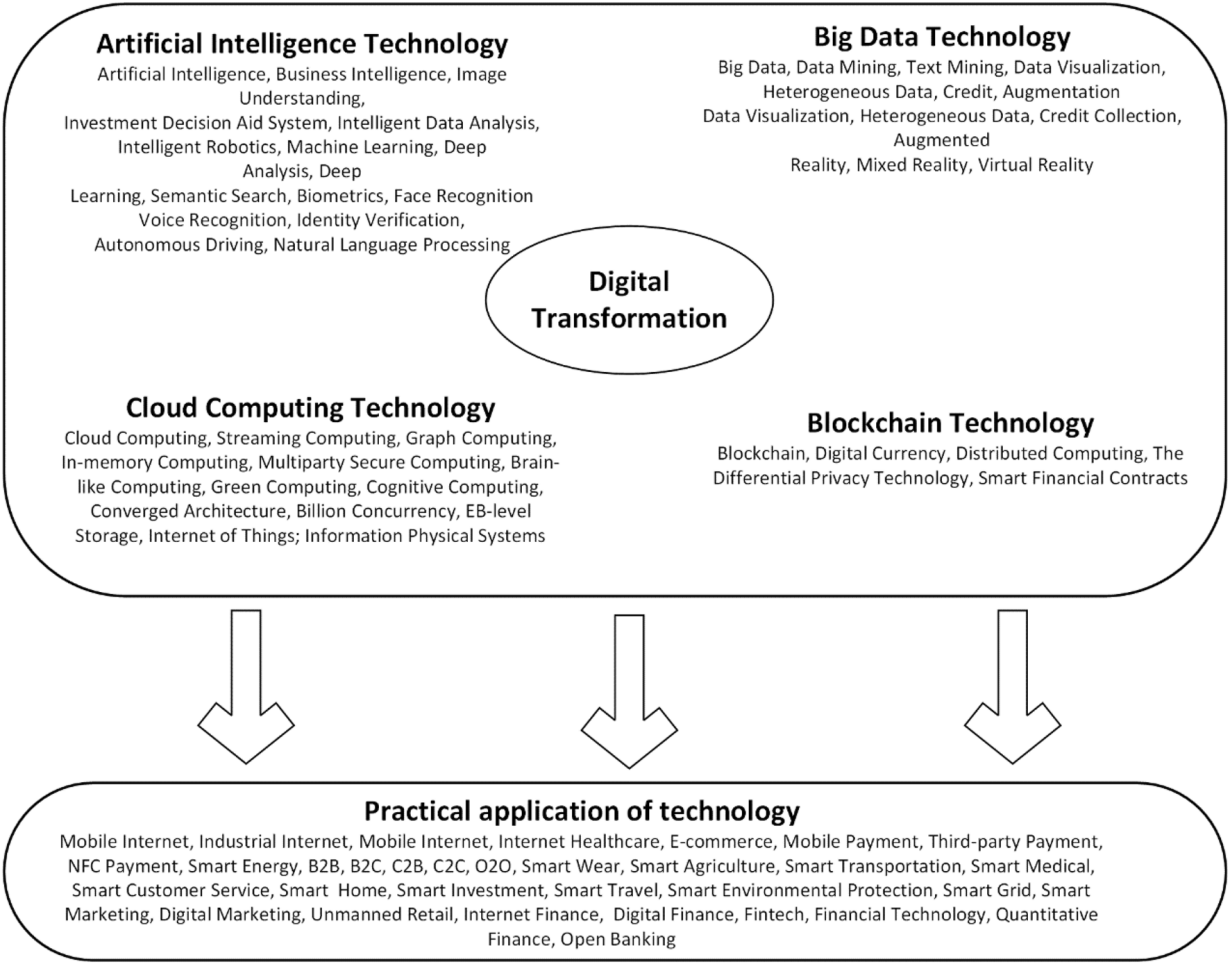
Structured feature word mapping of DT.

Digital transformation (DT): DT of enterprises involves not only the underlying digital technologies such as cloud computing, big data, blockchain, and artificial intelligence but also many other aspects of technology and business innovation. In this paper, we refer to the method of Fei et al.^44^ to statistically measure the corresponding keyword word frequencies in annual reports with five dimensions (digital technology, blockchain technology, cloud computing technology, big data technology, and artificial intelligence technology) and 76 related digital word frequencies to represent the degree of DT. In terms of variable design, this paper uses Python crawler technology to organize and collect the annual reports of A-share listed companies in Shenzhen and Shanghai exchanges. The Java PDFbox library is used to extract all the text content. These data serve as the data pool for feature word screening. In terms of identifying feature words for DT, this paper conducts sub-discussions in both academic and industrial fields. In terms of drawing on academic literature, this paper refers to a series of classic literature on the topic of DT^45,46^. In terms of important policy documents and research reports, we refer to *the Special Action Plan for Digital Empowerment of Small and Medium-sized Enterprises*, *the Implementation Plan for Fostering New Economic Development by Promoting the Action of "Going to the Cloud and Empowering Intelligence with Data"*, *the Digital Transformation Trends Report 2020*, and the recent *Government Work Report*, etc. The feature word database of DT is expanded, and structured classification is carried out according to the two aspects of underlying technology application and technical practice application, forming the feature word map in Fig. 2. On this basis, negative words ("no", "none", "didn't", etc.) are excluded, and the DT of non-company (including shareholders, customers, suppliers, and executive profiles of the company) is excluded. Finally, we extract data from the annual reports of listed companies through Python to search and match word frequency. The word frequency of key technology direction is classified and summarized to construct the index system of DT. Since this kind of data has typical "right bias" characteristics, this paper logarithmically processes them to obtain the overall indicators of DT.

#### Suppression variable

Earnings management (EM): Earnings management reduces the operational efficiency of firms and causes corporate decisions to deviate from the optimal situation, thus affecting ESG performance. Currently, there are two main approaches of real earnings management (REM) and accrued earnings management (AEM), which are represented by the absolute value of Roy-Chowdhury model results and the absolute value of manipulated accrued profits, respectively. The specific calculation formulae are as follows:

(1) REM: Referring to Roychowdhury^34^, Kim and Sohn^47^, we calculate the three items of abnormal cash flow from operating activities, abnormal discretionary expenses, and abnormal production costs, and then subtract the sum of the first two items from the abnormal production costs to obtain the indicator.

1 $$\frac{{CFO_{it} }}{{T_{it - 1} }} = \alpha_{0} + \alpha_{1} \frac{1}{{T_{it - 1} }} + \alpha_{2} \frac{{REV_{it} }}{{T_{it - 1} }} + \alpha_{3} \frac{{\Delta REV_{it} }}{{T_{it - 1} }} + \varepsilon_{it}$$

where *CFO*_*it*_ denotes the operating cash flow of firm $i$ in year $t$, $T_{it - 1}$ denotes the total assets of firm $i$ at the end of period $t-1$, *REV*_*it*_ represents the operating income of firm $i$ in year $t$, and accordingly $\Delta REV_{it}$ is its change, and $\varepsilon_{it}$ is the residual term of the model (abnormal operating cash flow).

2 $$\frac{{PROD_{it} }}{{T_{it - 1} }} = b_{0} + b_{1} \frac{1}{{T_{it - 1} }} + b_{2} \frac{{REV_{it} }}{{T_{it - 1} }} + b_{3} \frac{{\Delta REV_{it} }}{{T_{it - 1} }} + b_{4} \frac{{\Delta REV_{it - 1} }}{{T_{it - 1} }} + \varepsilon_{it}$$

*PROD*_*it*_ is the production cost of firm $i$ in period $t$, expressed as the sum of operating costs and inventory changes in the period, $\varepsilon_{it}$ is the abnormal production cost, and other variables are the same as in (1).

3 $$\frac{{DISEXP_{it} }}{{T_{it - 1} }} = c_{0} + c_{1} \frac{1}{{T_{it - 1} }} + c_{2} \frac{{REV_{it} }}{{T_{it - 1} }} + \varepsilon_{it}$$

*DISEXP*_*it*_ represents the manipulative costs, expressed as the sum of selling and administrative expenses, and the residuals represent the abnormal discretionary costs. The regressions of (1), (2), and (3) are performed by industry and year using firm data to obtain the three outliers required to calculate true earnings management.

4 $$TREM_{it} = ( - 1)U\_CFO_{it} + U\_PROD_{it} + ( - 1)U\_DISEXP_{it}$$

*TREM*_*it*_ denotes true earnings management, $U\_CFO_{it}$, $U\_PROD_{it}$, $U\_DISEXP_{it}$ indicates abnormal operating cash flows and abnormal production costs, and abnormal discretionary expenses, respectively.

(2) AEM: In this paper, the modified Jones model proposed by Dechow et al.^48^ is used to calculate manipulable accrued profits:

5 $$\frac{{TA_{it} }}{{T_{it - 1} }} = \theta_{0} \frac{1}{{T_{it - 1} }} + \theta_{1} \frac{{\Delta REV_{it} }}{{T_{it - 1} }} + \theta_{2} \left( {\frac{{PPE_{it} }}{{T_{it - 1} }}} \right) + \varepsilon_{it}$$

6 $$NDA_{it} = \hat{\theta }_{0} \frac{1}{{T_{it - 1} }} + \hat{\theta }_{1} \frac{{\Delta REV_{i,t} - \Delta REC_{i,t} }}{{T_{it - 1} }} + \hat{\theta }_{2} \left( {\frac{{PPE_{i,t} }}{{T_{i,t - 1} }}} \right)$$

where $TA_{it}$ denotes the total accrued profit, *PPE*_it_ is the net value of fixed assets of firm $i$ in year $t,$ *NDA*_*it*_ denotes the unmanageable accrued profit, $\Delta REC_{it}$ represents the change in accounts receivable, and other variables are consistent with the above. By fitting formula (5), $\hat{\theta }_{0}$, $\hat{\theta }_{1}$, $\hat{\theta }_{2}$ are obtained. Parameters are substituted into Formula (6) to calculate the non-manipulable accrued profit. After that, the total accrued profit and the unmanageable accrued profit are substituted into (7) to find the manipulable accrued profit.

7 $$DA_{it} = \frac{{TA_{it} }}{{T_{it - 1} }} - NDA_{it}$$

### Quantitative approach: model construction and control variables

#### Benchmark regression model

This paper constructs an econometric model based on the panel data to analyze the effects of DT and EM on corporate ESG performance, and the model is set as follows:

8 $${ESG}_{it}={\upsilon }_{0}+{\upsilon }_{1}\times DT{ }_{it}+{\upsilon }_{2}\times {Z}_{it}+{\gamma }_{i}+{\rho }_{t}+{\mu }_{it}$$

where ${ESG}_{it}$ denotes enterprise ESG performance; $DT{ }_{it}$ denotes the degree of digital transformation; ${Z}_{it}$ denotes the control variable; ${\upsilon }_{0}$ is the intercept term, ${\upsilon }_{1}, {\upsilon }_{2}$ are the coefficient parameters corresponding to the explanatory variables and each control variable, respectively; ${\rho }_{t}$ denotes the time effect that does not change with individuals, ${\gamma }_{i}$ denotes the individual effect that does not change with time, and ${\mu }_{it}$ is the random disturbance term.

#### Suppression effect model

The suppression effect and the mediating effect are both indirect effects. Some scholars consider the suppression effect as a broad mediating effect. When the effect of the core explanatory variable on the dependent variable is partially masked by the indirect effect of the mediating variable. The suppression effect is determined when the direct and indirect effects act in opposite directions^49^. In this paper, EM is used as a suppression indicator, which is divided into AEM and REM. Also, the following model is constructed to test whether DT affects ESG through the suppression effect of EM.

9 $$AEM={\upsilon }_{0}+{v}_{3}\times DT{ }_{it}+{\upsilon }_{2}\times {Z}_{it}+{\gamma }_{i}+{\rho }_{t}+{\mu }_{it}$$

10 $${REM}_{it}={\upsilon }_{0}+{v}_{3}\times DT{ }_{it}+{\upsilon }_{2}\times {Z}_{it}+{\gamma }_{i}+{\rho }_{t}+{\mu }_{it}$$

11 $${ESG}_{it}={\upsilon }_{0}+{\upsilon }_{4}\times DT{ }_{it}+{v}_{5}\times AEM{ }_{it}+{\upsilon }_{2}\times {Z}_{it}+{\gamma }_{i}+{\rho }_{t}+{\mu }_{it}$$

12 $${ESG}_{it}={\upsilon }_{0}+{\upsilon }_{4}\times DT{ }_{it}+{v}_{5}\times REM{ }_{it}+{\upsilon }_{2}\times {Z}_{it}+{\gamma }_{i}+{\rho }_{t}+{\mu }_{it}$$

where ${v}_{1}$ is the total effect, ${v}_{3}{v}_{5}$ is the indirect effect, and ${v}_{4}$ is the direct effect. Based on Eq. (8), Eqs. (9) and (10) are estimated to test whether the effect of DT on EM is significant. If the regression result is significant, it means that DT affects EM. Finally, the regressions of Eqs. (11) and (12) are performed. If the coefficient of EM is significant and ${v}_{3}{v}_{5}$ is in the opposite direction of ${v}_{4}$, it indicates that there is a suppression effect of EM in the impact of DT on ESG.

Based on the existing literature, we also use the following control variables and the descriptive statistics of the overall data are shown in Table 1:

**Table 1:** Descriptive statistics of variables.

Variable	Obs	Mean	Std. Dev	Min	Max
ESG	15,134	6.593	1.172	1	9
DT	15,134	1.088	1.385	0	6.265
AEM	15,134	0.073	0.128	0	3.261
REM	15,134	0.141	0.189	0	7.128
ES	15,134	22.628	1.405	17.779	28.635
BS	15,134	2.234	0.243	1.099	3.296
SOC	15,134	0.938	1.398	− 9.210	4.027
ALR	15,134	0.477	0.198	0.007	0.993
ROA	15,134	0.038	0.456	-27.595	1.117

Enterprise size (ES): To some extent, enterprise size reflects the degree of concentration of resources, labor, and means of production in an enterprise. Existing studies have concluded that there is a significant effect of enterprise size on sustainable development^50^. This paper uses the logarithm of the enterprise's total assets at the end of the year to represent the size.

Board size (BS): The board of directors, as the decision-making body of the enterprise, has a significant impact on the strategy and operation of the enterprise. It can directly affect the level of sustainability through decision science^51^. In this paper, the logarithm of the number of board members is used to represent this indicator.

Separation of ownership and control (SOC): The degree of separation of the two rights can reflect the separation between the ownership of capital and the operation of capital. It is generally believed that when the separation of the two rights is large, the effective controller has the power and incentive to encroach on the company's interests, thus affecting the sustainability of the company^52^. Therefore, the logarithm of the difference between the control and ownership rights owned by the controller is used to represent this indicator.

Asset-liability ratio (ALR): The asset-liability ratio reflects the ratio of total assets of an enterprise to liabilities. It can reflect the comprehensive debt level of enterprises. An enterprise's assets and liabilities can directly affect its accountability capital adequacy, and thus its ESG performance^53^. The ratio of total liabilities to total assets is used to represent this indicator.

Return on assets (ROA): Return on assets can effectively measure the utilization of assets and reflect the business management level of the enterprise. Enterprises with higher returns on assets tend to have more resources to support ESG investments as well as fulfill social responsibilities^54^. Therefore, return on net assets is used to represent this indicator.

### Qualitative approach: Variable selection and calibration

With the fsQCA analysis, we aim to fill the research gap related to ESG performance with a new methodology, data, and results. It remedies the possible bias of the econometrics methodology while analyzing the paths and patterns of the impact of DT and EM on driving ESG performance. It can provide guidance and reference for how enterprises can rely on DT to promote ESG in the context of digitalization. Qualitative Comparative Analysis (QCA) was first proposed by sociologist Ragin. The method is based on holism, which is grounded in the multiple causal relationships between the overall grouping and the outcome. First, the fsqca approach considers that first-order elements are concurrent and that various higher-order groupings may have equifinality for outcomes^55^. Therefore, it is possible to identify equivalent groupings and thus identify complementary substitution relationships between conditions. Second, fsqca can answer the asymmetry problem. In combination with practice, the conditions that lead to higher and lower ESG performance are not opposed to each other, which applies to fsqca's answer to the asymmetry question. Finally, the fsqca method can fully combine the advantages of qualitative and quantitative approaches, combining the uniqueness and depth of a case in traditional qualitative analysis with the external generalizability in quantitative analysis. The method applies to large, medium, and small case studies^56,57^.

**Figure 3:**
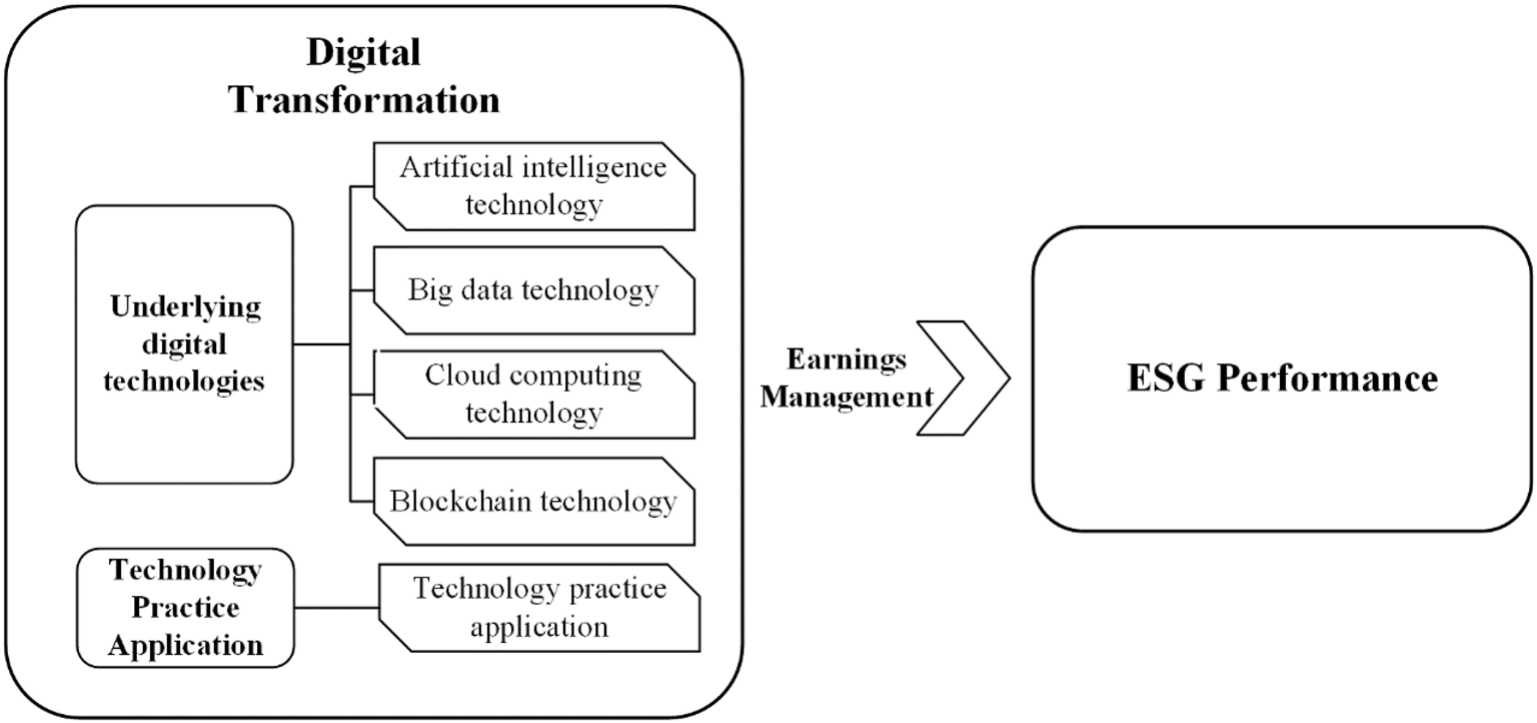
Configuration conditions chart.

Due to the differences in the impact of different aspects within DT on ESG, DT is specifically divided into underlying digital technologies and technology practice applications. Among them, the underlying digital technologies include artificial intelligence technology (AIT), which reflects the underlying digital intelligence; big data technology (BDT), which represents the underlying digital resources; cloud computing technology (CCT), which represents the underlying digital devices; blockchain technology (BT), which represents the underlying digital information, and technology practice application (TPA), which includes digital technology application. The same text analysis method is used for keyword frequency measurement. Meanwhile, the conditional element of EM is incorporated to reflect management performance manipulation. To ensure an in-depth analysis of the sample cases, fully explain the complex causes of the results, and explore the current DT driving path to ESG, the latest year 2021 is selected as the sample period for qualitative analysis in this paper. It not only meets the similarity and diversity of sample cases, but also the sample age is relatively new, which can better reflect the specific path of current DT to drive ESG performance. It is of more practical significance and reference significance for the current DT to promote the sustainable development of enterprises. The specific variables are selected as in Fig. 3.

### Ethics approval and consent to participate

This study involves the macro data of the human economy and society. All the data are from the China Stock Market Accounting Research (CSMAR), as well as the relevant corporate annual report. The data collection process is in line with the ethical and moral standards. The research method of this study is fsqca and spatial econometrics, and there is no need for ethical approval and animal experiment content. The author guarantees that the process, content, and conclusion of this study do not violate the theory and moral principles.

## Results and discussion

### Results of the quantitative approach

#### Benchmark regression analysis

Table 2 shows the mixed OLS regression, random effects regression, and fixed effects regression with ESG performance as the explanatory variable. The regression results of all three econometric models indicate that the degree of DT can significantly reduce ESG performance, supporting hypothesis 1. Based on the results of the F-test, and Hausman test, the fixed-effects model is the optimal model. Specifically, the impact coefficient of the degree of DT is − 0.013, which is significant at the 5% level. DT is accompanied by the integration of digital technologies with business activities. Through DT, we can improve the efficiency of the organization's operation, refine user needs, and improve the long-term operation of the enterprise. However, DT is a long-term process. According to the “IT paradox”, DT needs to be carried out gradually in the context of the enterprise's development, industry conditions, and planning. Digital change implies a high degree of uncertainty, which can add some hidden costs to the enterprise. The introduction of digital technology and its skilled application also require additional management costs. At the same time, DT requires a large amount of innovation investment and long-term factor investment. According to the dynamic capability theory, enterprises face a complex and changing external environment, which will have a certain impact on internal systems during DT. ESG performance incorporates environmental responsibility and social responsibility as opposed to traditional performance evaluation. At the same time, the performance evaluation of corporate governance is also based on the sustainability evaluation of stakeholders. Influenced by self-interest behavior, enterprises tend to ignore environmental and social responsibility when facing the adjustment of DT and tend to maintain the interests of owners more. This results in a lack of investment in external non-operational indicators of enterprises, leading to a significant decline in ESG performance. This phenomenon further reflects that the DT of Chinese enterprises is not yet complete and not deeply integrated with the operational aspects. In terms of control variables, both ES and SOC are found to be able to cause a significant positive impact on ESG performance. Specifically, the coefficient of the effect of ES on ESG is 0.218, which is significant at the 1% level. The increase in size facilitates the formation of economies of scale and lower marginal costs. When purchasing raw materials, enterprises can also expand the purchase volume, which is conducive to reducing production costs and alleviating business pressure. It is conducive to improving the ability of enterprises to fulfill their responsibilities and driving the fulfillment of their social and environmental responsibilities. The coefficient of the effect of SOC on ESG is 0.027, which is significant at the 1% level. The expansion of the SOC will increase shareholder control gains but does not affect ESG performance. It indicates that the effective controlling shareholders will still focus on non-financial performance and on improving the corporate image as well as the future development space. BS and ALR can reduce ESG. At the 1% level, the impact coefficient of BS is − 0.141 and the impact coefficient of ALR is − 0.797. The increase in BS leads to diversification and decentralization of decision-making. Due to different backgrounds, industries, and understandings, the investment efficiency of non-financial performance is reduced, which leads to the decline of ESG performance. The increase in ALR weakens the solvency of enterprises and reduces their productivity of enterprises. As a result, enterprises pay less attention to sustainable development and other external performance, resulting in the decline of ESG performance.

**Table 2:** Benchmark regression results.

Model	ESG		
	1 OLS	2 RE	3 FE
DT	− 0.018*** (− 2.83)	− 0.013*** (− 2.73)	− 0.013** (− 2.54)
ES	0.419*** (50.95)	0.335*** (28.19)	0.218*** (12.02)
BS	0.001 (0.04)	− 0.097*** (− 2.87)	− 0.141*** (− 3.86)
SOC	0.005 (0.85)	0.018** (2.59)	0.027*** (3.27)
ALR	0.921*** (16.98)	− 0.825*** (− 13.71)	− 0.797*** (− 11.19)
ROE	0.061*** (3.28)	0.026* (1.82)	0.018 (1.19)
Obs	15,134	15,134	15,134
R^2^	0.26	0.30	0.11
F test			7.39***
LM test		13,045.22***	
Hausman test			347.42***
Year	Control		
Industry	Control		

*, **, *** indicate significance at the 10%, 5%, and 1% levels, z-statistics are shown in parentheses.

#### Suppression effect analysis

According to the theoretical analysis, DT can reduce corporate information asymmetry by influencing the adjustment of EM, which ultimately affects ESG performance. To test this mechanism, AEM, and REM are selected as suppression variables to empirically analyze the suppression transmission mechanism, respectively. According to the results in Table 3, DT can significantly reduce AEM with an impact coefficient of − 0.196, while the effect on REM is not significant. The potential reason is that DT improves the disclosure of internal business information and increases the communication of information between internal and external companies. Relying on digital technology, the internal data generated from business operations are analyzed and transmitted promptly, and the degree of external informatization increases. Compared to traditional operations, DT facilitates the timeliness and effectiveness of corporate information disclosure, while enabling increased visibility of the production chain. In addition, DT enables dynamic real-time monitoring of operations and feedback on risks and returns in a complex and volatile external environment. It reduces information asymmetry and reduces the room for management to adjust accounting information and manipulate AEM, leading to a decline in AEM. REM is more difficult to detect than AEM because it is more insidious. AEM usually uses accounting treatment to conceal management's performance issues. Common methods include the use of accounting policy changes and choices to abuse fair value and asset impairment accrues and reversals, etc. This type of EM generally requires the attachment of relevant information to the accounting statements, and it is difficult to distinguish REM from daily operating activities. The period of digital transformation has brought about an impact of digital technology on the original production model, making it more difficult to identify REM. Meanwhile, the greater flexibility in the timing and space of activities of REM further reduces the significance of the impact on it. Column (3) in Table 3 analyzes the suppression mechanism of DT on ESG performance using AEM as a suppression variable. The results show that the coefficient of the effect of AEM on ESG is −  0.109, which is significant at the 5% level. The coefficient of the impact of DT on ESG is − 0.009, respectively, which does not pass the significance test.

**Table 3:** Suppression model regression results.

FE-Model	AEM	REM	ESG
DT	− 0.196* (− 1.86)	0.043 (0.55)	− 0.009 (− 1.53)
AEM			− 0.109** (− 1.97)
ES	0.035 (0.79)	0.034 (0.75)	0.233*** (9.96)
BS	0.091 (1.15)	0.091 (1.14)	− 0.112*** (− 2.68)
SOC	0.030 (1.58)	0.028 (1.51)	0.033*** (3.37)
ALR	− 0.162 (− 0.95)	− 0.173 (− 1.02)	− 0.769*** (− 8.65)
ROE	− 0.028 (− 0.80)	− 0.031 (− 0.87)	0.058*** (3.17)
Obs	15,134	15,134	15,134
R^2^	0.09	0.09	0.06
F test	1.43***	1.43***	7.51***
Hausman test	91.90*	93.69*	300.86***
Year	Control		
Industry	Control		

*, **, *** indicate significance at the 10%, 5%, and 1% levels, z-statistics are shown in parentheses.

Combined with the previous findings, it is found that the indirect effect result (− 0.196*− 0.109) is opposite to the result of the direct effect (− 0.009). It indicates that DT has a negative effect on enterprise ESG, while DT can constrain AEM, thus the direct negative effect of DT on ESG is suppressed by the indirect positive effect of DT in suppressing AEM. Hypothesis 3 is supported. The possible reason is that AEM, as a means of performance manipulation by the management, reduces the quality of accounting statements, which is not conducive to the corporate image and business stability. The increase in AEM reflects the decline of the management's internal governance mechanism and moral constraints, and the decline in the fulfillment of non-performance responsibilities such as corporate social responsibility, environmental responsibility, and internal responsibility. Therefore, AEM has a significant negative effect on ESG. The introduction of digital technology enables a large amount of data to be collected and collated, which can significantly improve information transparency. The space of EM will be limited, making the financial statements more truthful and reliable, thus restraining the manipulation of performance behavior. The asymmetry of information is gradually reduced, the speculative activities of the management for personal gain are reduced, and the long-term development capability is enhanced, and thus the adverse impact of DT on the ESG performance is reduced. That is, DT restricts AEM, thus mitigating the negative impact on ESG. In the suppression model, the results of each control variable are largely consistent with the previous findings, reflecting the relatively stable effects of each control variable on ESG performance. Specifically, BS and SOC have significant pull effects on ESG, with impact coefficients of 0.233 and 0.033, respectively. BS and ALR still have a negative impact on corporate sustainability. The expansion of the number of directors has led to lower awareness of corporate responsibility, further reflecting the difference in the level of attention given to sustainable development. In the future, there is a need to strengthen the promotion and guidance of the ESG concept. The increase in the ALR reflects the increase in debt financing, which leads to higher operating pressure and reduces the ESG level. Meanwhile, the significance of ROA has increased, with an impact coefficient of 0.058, which is significant at the 1% level. The increase in investment returns drives the efficiency of internal investment operations and thus the capital reserve for ESG investment increases. It further verifies that when internal operational pressure is reduced, firms will pay more attention to the needs of other stakeholders and increase responsible investment.

#### Robustness test

The robustness test aims to assess the stability of the explanatory power of evaluation methods and indicators. It verifies that they can still provide stable evaluation results when some parameters are changed. To further analyze the model’s robustness, the replacement variable method is chosen in this paper. To explore the influencing factors of ESG performance in-depth, replacement explanatory variables are selected. A more detailed caliber breakdown of DT is performed based on technology composition and application. And the regression analysis is re-run. Referring to Zhao^58^, the DT indicator is constructed by counting the frequencies of 99 digitization-related words in four dimensions, including digital technology applications, Internet business models, smart manufacturing, and modern information systems. The specific word frequency information is shown in the Appendix Figure S1. The regression results are shown in Table 4, and the results show that the values of these coefficients do not change much and there is no change in direction and significance, and the empirical results in this section are robust.

**Table 4:** Robustness test.

Model	OLS	RE	FE
DT-B	− 0.044*** (− 6.85)	− 0.023*** (− 4.65)	− 0.008 (− 1.49)
Control variables	Yes	Yes	Yes

*, **, *** indicate significance at the 10%, 5%, and 1% levels, z-statistics are shown in parentheses.

#### Endogeneity test

The endogeneity test is performed considering the possible existence of the policy and institutional factors within the residuals, producing problems such as omitted variables. Two-stage least squares analysis is performed using instrumental variable IV estimation. The instrumental variables are subject to both exogeneity and correlation assumptions. Exogeneity requires that the instrumental variables are independent of the perturbation term. Correlation requires that it is related to and affects the explanatory variables only through the endogenous variables. The instrumental variables have a strong historical nature, therefore, the perturbation terms in the sample period cannot affect the instrumental variables and satisfy the independence condition. Referring to Zhong et al.^59^, the independent variables are determined with one period lag in the past and are independent of the current period perturbation term, and thus can be used as instrumental variables. Column (1) in Table 5 shows the regression results of the instrumental variables on DT. The regression coefficient is 0.265, which is significant at the 1% level, indicating a significant positive effect of instrumental variables on DT. Column (2) reports the results of the two-stage IV regression when the explanatory variable is ESG. The regression coefficient for DT is significant at the 1% level of − 0.145. The results are generally consistent with the benchmark regression. Hausman's test result is 38.60, which is significant at the 1% level. Therefore, in this case, it is a reasonable approach to reject the original hypothesis and apply the instrumental variables approach. In summary, the robustness of the basic findings is further verified after considering the endogeneity issue.

**Table 5:** Regression results of instrumental variable.

	OLS(DT)	SLS(ESG)
DT		− 0.145*** (− 4.99)
IV: L.DT	0.265*** (28.07)	
ES	0.048*** (4.39)	0.389*** (42.61)
BS	− 0.144*** (− 2.07)	0.020 (0.47)
SOC	− 0.002 (− 0.21)	− 0.003 (− 0.47)
ALR	− 0.236*** (− 2.99)	− 0.787 *** (− 12.07)
ROE	− 0.081*** (− 2.60)	0.129*** (5.01)
Obs	11,293	11,293
R^2^	0.07	0.15
Hausman test		38.60***

*, **, *** indicate significance at the 10%, 5%, and 1% levels, z-statistics are shown in parentheses.

### Results of the qualitative approach

The previous study shows that there is a direct effect of DT on ESG performance and a suppression effect of AEM in the path. The effect of REM is not significant, thus it is more reasonable to use AEM as a suppression variable to reflect performance manipulation. Therefore, AEM is used as one of the conditional variables. The specific driving path of DT and EM to promote ESG performance improvement is analyzed.

#### Variable calibration

The previous study shows that there is a direct impact of DT on ESG performance, a suppression effect of AEM in the impact path, and an insignificant effect of REM. Therefore, AEM is used as one of the conditional variables to analyze the specific driving paths of DT and EM on ESG performance. This paper uses a direct calibration method. The 95th percentile, median, and 5th percentile of the conditional and outcome samples are set as completely affiliated, intersection, and completely unaffiliated points, respectively. The advantage of using the median rather than the mean is that the median is less sensitive to outliers^60^. At the same time, due to technical deficiencies in some numerical techniques, the median is not differentiated from the 5th percentile. Therefore, this category of variables is artificially set to be fully affiliated with 1 and fully unaffiliated with 0. The calibration results are shown in Table 6.

**Table 6:** Variable calibration results.

Condition	Completely affiliated (95th percentile)	Intersection (median)	Completely unaffiliated (5th percentile)
ESG	9	7	5
AIT	1	–	0
BDT	18.75	1	0
CCT	1	–	0
BT	1	–	0
TPA	24.75	2	0
AEM	0.159	0.038	0.004

#### Necessity analysis

Before conducting a sufficiency study, it is necessary to test whether the condition variable becomes a necessary condition for the outcome variable. A necessary condition is a bottleneck that constrains the outcome. When a condition is always present when the outcome occurs, that condition becomes a necessary condition. At the technical means level, certain necessary conditions may be eliminated as logical residuals and do not appear in the parsimonious or intermediate solutions, which can be biased for generating results. Therefore, each conditional variable should be analyzed before the group analysis to see if it constitutes a necessary condition. In this paper, we test whether a single condition (including its non-set) constitutes a necessary condition for a high valuation of ESG performance in 2021. (Consistency greater than 0.9 is a necessary condition). The results of the test are presented in the following table: as shown in Table 7, there is no univariate necessary condition for a high valuation of ESG performance.

**Table 7:** Necessity test results.

2021	ESG	
Condition tested	Consistency	Coverage
AIT	0.42	0.50
~AIT	0.58	0.48
BDT	0.51	0.66
~ BDT	0.73	0.57
CCT	0.46	0.50
~ CCT	0.54	0.48
BT	0.15	0.53
~ BT	0.85	0.48
TPA	0.56	0.66
~ TPA	0.70	0.59
AEM	0.60	0.62
~ AEM	0.67	0.64

#### Adequacy analysis

Considering asymmetric causality, this paper uses the fsqca method to analyze DT and AEM configurations that produce high ESG performance. At the same time, this paper qualitatively analyzes and names the discovered states to deepen the configuration theory. Thus, it can help enterprises achieve high ESG performance modes through DT and EM. When conducting configuration analysis (adequacy analysis), this paper sets the threshold of case frequency as 3, the original consistency as 0.75, and the PRI consistency threshold as 0.45. The truth value table is shown in Appendix Table S1. To synthesize the simple solution, intermediate solution, and complex solution, this paper chooses to sketch the path diagram to represent the feasible path. If there are the same core conditions and different edge conditions, two different policy paths in the same path are selected for naming (S1a, S1b). At the same time, different path names (S1, S2, S3) are carried out for different core conditions, and different configuration characters are named according to different path conditions.

Table 8 is the configuration path analysis of the high valuation group of ESG performance in 2021. A total of 6 configurations are obtained by the high valuation group, and these 6 configurations are sufficient conditions to promote the improvement of ESG performance. The consistency of the overall configuration is 0.7091, indicating that in the cases of the six configurations, 70.91% of listed enterprises' ESG performance has been improved. The coverage of the total configuration is 0.5691, indicating that the six configurations together explained 56.91% of the cases. Based on six conditional configuration paths, the influence of each conditional variable on ESG performance high valuation is further analyzed. The core condition of configuration S1 is the application of digital technology and the absence of AEM. Among them, the peripheral condition of configuration S1a is the absence of BT, and the peripheral condition of configuration S1b is the existence of AIT, BDT, and CCT. The consistency is 0.3456 and 0.1668, respectively, covering 35 cases. The core condition of configuration S2 is the existence of AIT, the absence of CCT and AEM, and the peripheral condition is the absence of BT. The consistency of this configuration is 0.0585, covering 20 cases. The configuration of S3 is driven by four peripheral conditions, namely the existence of AI and BDT, BT, and the absence of AEM. The consistency of this configuration is 0.1560, covering 20 cases. The core condition of the S4 configuration is the existence of BDT, the absence of CCT, and the peripheral condition is the absence of BT. The consistency of this configuration is 0.1715, covering 18 cases. The core condition of configuration S5 is the absence of AIT and CCT and the existence of TPA. The consistency of this configuration is 0.1753, covering 20 cases. The core condition of configuration S6 is the existence of AIT and AEM, and the absence of BT and TPA. The consistency of this configuration is 0.1423, covering 20 cases.

**Table 8:**
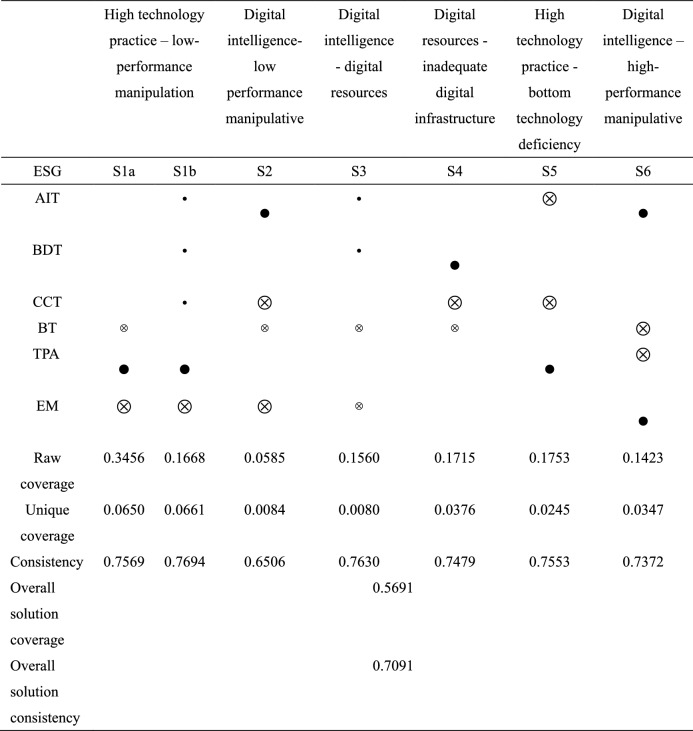
Analysis of the configuration.

Note:  denotes the presence of core conditions;  denotes the absence of a core condition;  denotes the presence of peripheral conditions;  denotes the absence of peripheral conditions; blank cells represent irrelevant (“don’t care”) conditions which may be either present or absent in a configuration.

#### Robustness test

The threshold setting of the QCA method has a certain flexibility, and the analysis results may change with the threshold, so it is necessary to conduct a robustness test on the results. This paper adopts the method of adjusting the frequency threshold and consistency threshold to test robustness. When adjusting the consistency threshold, it is found that the number of configurations in the truth table analysis is affected, so we raise the consistency threshold to 0.8 and reprocessed the sample data^61^. The results show that even when the threshold is adjusted, the condition configuration is consistent and no contradictory results appear, so our research conclusion is robust. The specific results are shown in Table 9.

**Table 9:**
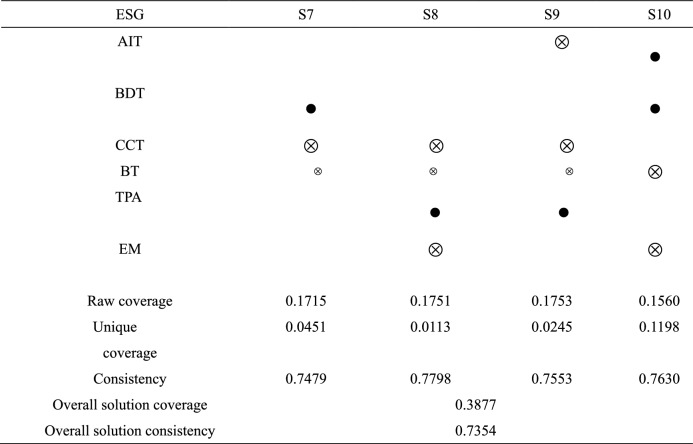
Robustness test.

Note:  denotes the presence of core conditions;  denotes the absence of a core condition;  denotes the presence of peripheral conditions;  denotes the absence of peripheral conditions; blank cells represent irrelevant (“don’t care”) conditions which may be either present or absent in a configuration.

#### Path and model analysis

**Figure 4:**
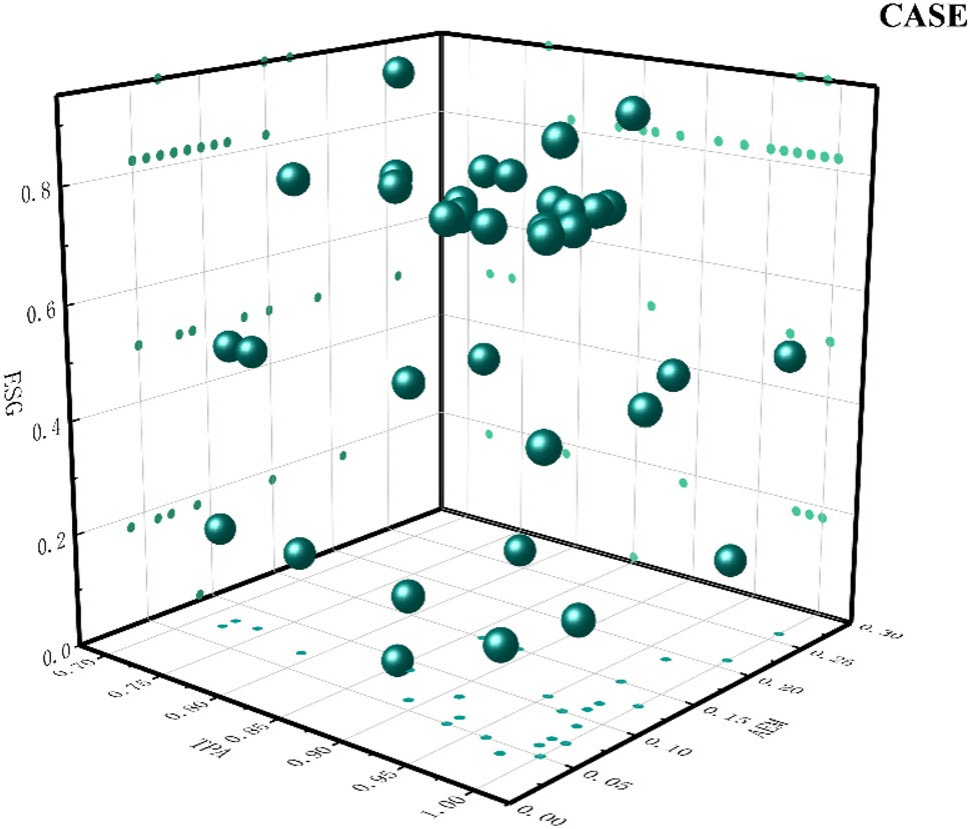
Path 1 case diagram.

Path 1: TPA* ~ EM* ~ BT and TPA* ~ EM*AIT*BDT*CCT are the high valuations of ESG performance driven by a high level of digital technology usage with low-performance manipulation behavior. Some of these companies lack BT, while others have a high level of AIT, BDT, and CCT. This category of enterprises mainly relies on DT and deep integration with core market tasks to update the technology of specific scenarios in the economy and society, thus forming a new business model. The use of digital technology emphasizes companies' reliance on digital technology to generate effective innovation outputs and applications in the marketplace. By integrating digital technology with complex business scenarios, the technology chain within the enterprise is gradually transferred to the external front-end market applications. At the same time, such companies do not use information asymmetry to manipulate earnings when enhancing their digital technology practices but reflect the actual operating conditions of the company truly and concretely. Improving the level of trust of external investors in the enterprise. Combined with the peripheral conditions, it is found that some enterprises have insufficient BT, reflecting the inadequacy of digital information technology. BT is difficult to tamper with and decentralized, which provides great convenience for enterprises to store data information. Xu et al. established a K-Out-of-M candidate model by using blockchain technology and introduced the APV algorithm to alleviate the problems of lack of anonymity, excessive concentration, and easy forgery of data. However, it is affected by storage costs and privacy issues, which reduces the applicability of enterprises^62^. Xu and He adopted the Latent Dirichlet Allocation theme model and found that there are certain technical challenges in blockchain technology. Enterprises often seek to hide transaction data, resulting in a lack of trust among participants and making the implementation of blockchain technology difficult^63^. Other enterprises focus on investment in digital intelligence, digital resources, and digital devices to continuously improve the development of underlying digital technology and jointly promote the progress of ESG performance. The enterprises that fit this model are shown in Fig. 4.

**Figure 5:**
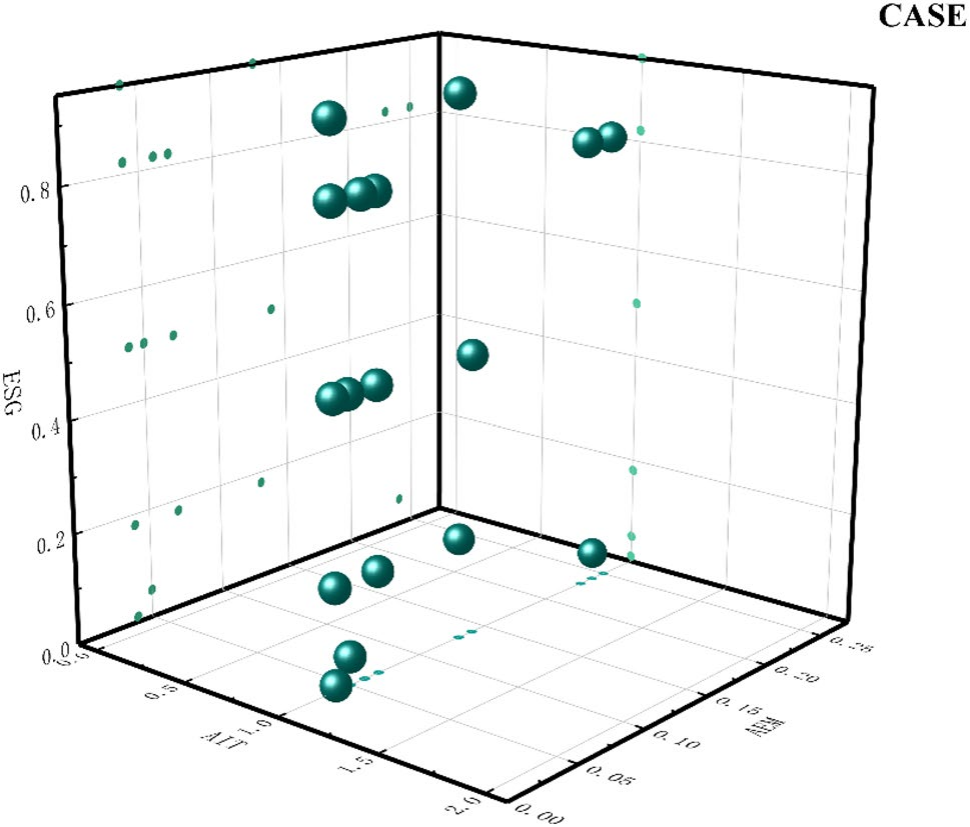
Path 2 case diagram.

Path 2: AIT* ~ CCT* ~ EM* ~ BT are CCT-deficient enterprises relying on AIT with low-performance manipulation behaviors to drive ESG performance. This type of enterprise BT is also deficient. The lack of CCT reflects the lack of development of enterprise digital devices and the poor computing power of data. Taking the Internet of Things (IoT) technology as an example, the widespread application of LOT technology has generated a huge amount of sensor data involving both normal and abnormal data, and the abnormal situations involved may have a great impact on the sustainability of the industry^64^. Faced with this situation, such enterprises rely on AIT to bring into play the decision-making power of digital intelligence. According to the dynamic capability theory, in a complex competitive environment, it is important to develop dynamic capabilities based on the differences of individual firms. Mikalef and Gupta^65^ argue that heterogeneous resources constructed through artificial intelligence can enhance the level of innovation dynamics and performance of firms. Kar et al. argue that artificial intelligence significantly contributes to the ESG performance of firms. The adoption of AI technologies can improve enterprises' efficiency, reduce polluting energy use, and promote the development of the circular economy^66^. At the same time, this category of enterprises continues to improve external trust, reduce performance manipulation, and promote ESG performance. The indirect transmission mechanism of AEM on ESG performance is further verified. The enterprises that fit this model are shown in Fig. 5.

**Figure 6:**
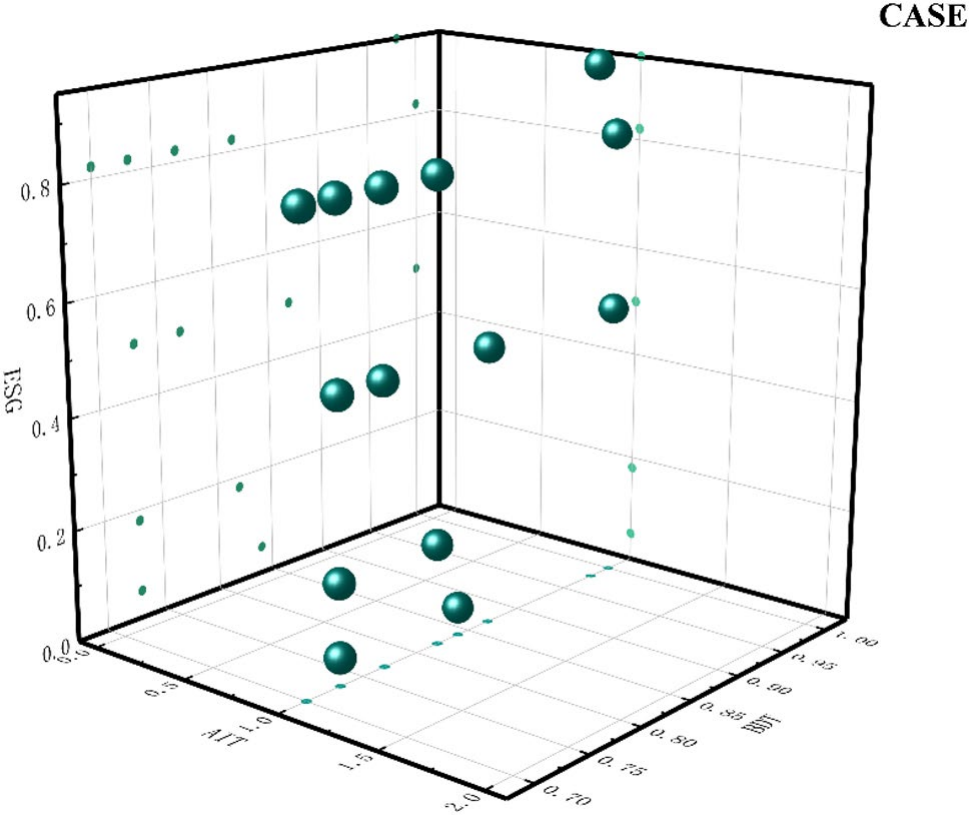
Path 3 case diagram.

Path 3: AIT*BDT* ~ BT* ~ EM relies on AIT and BDT to improve ESG. This group of enterprises suffers from the lack of BT and low AEM behavior. The configuration enterprise uses BDT to organize massive amounts of data and aggregate huge amounts of information. The core digital information is extracted quickly, processed, and organized into digital resources that can improve business operations. It effectively improves the efficiency of business operations and supports the fulfillment of external responsibilities such as social responsibility. Based on organizing massive resources with BDT, relying on digital intelligence, and linking upstream and downstream partners to build a green trade chain. Digital intelligence can also enhance the science of corporate decision-making. Technologies such as data analytics, neural networks, and knowledge graphs are integrated to build a beneficial information system for business operations^67^, thus improving the sustainability of the enterprise. The lack of BT further validates the general lack of blockchain investment in listed companies, and the issues of immutability, irrevocability, and lack of privacy lead to the lack of application of this technology. In addition, low EM behavior of management also indirectly affects ESG performance as a peripheral condition. The enterprises that fit this model are shown in Fig. 6.

**Figure 7:**
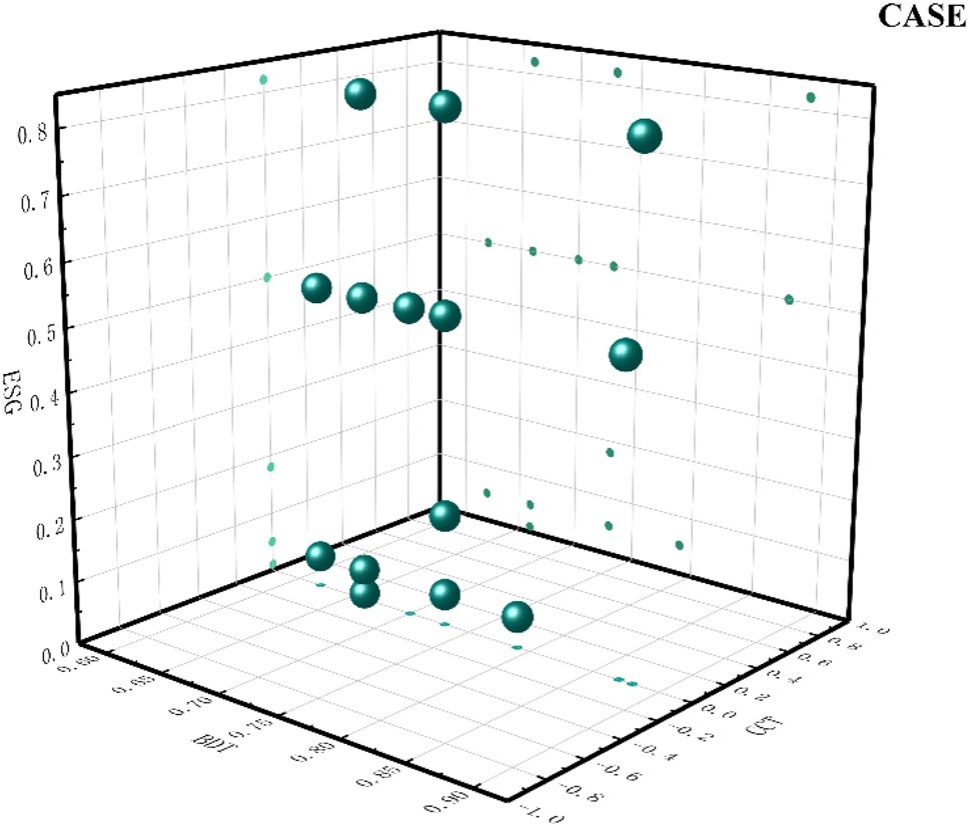
Path 4 case diagram.

Path 4: BDT* ~ CCT* ~ BT is the enterprise with insufficient CCT relying on BDT to drive ESG. Such enterprises also have a lack of BT. This type of enterprise has poor development of underlying digital technology, and there is a lack of digital equipment and digital information. Faced with the lack of development of the other three digital technologies, enterprises continue to pay more attention to the digital resources of big data. To fully exploit the information in the massive data, explore the value in the data, and rely on the advantages of digital resources to drive the sustainable development capability of enterprises. The enterprises that fit this model are shown in Fig. 7.

**Figure 8:**
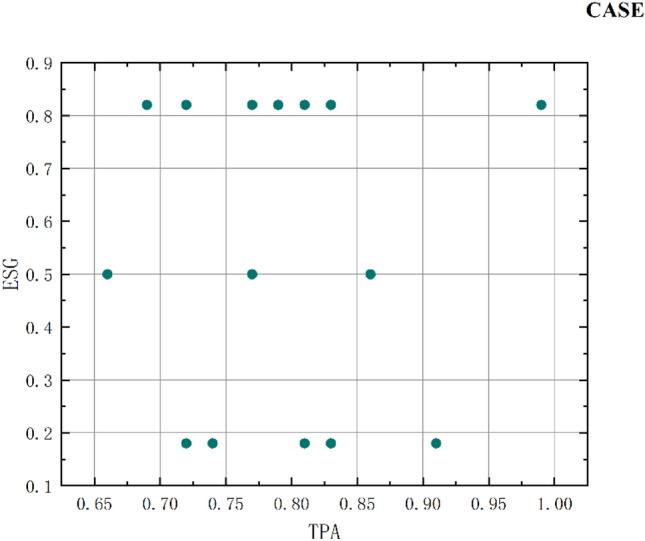
Path 5 case diagram.

Path 5: TPA* ~ AIT* ~ CCT are enterprises with insufficient AIT and CCT relying on the use of digital technology to drive ESG performance. This group of enterprises has insufficient digital infrastructure construction and lacks core underlying digital technology advantages. It mainly reflects the lack of digital intelligence and digital devices. As a result, the impact of digital technology on traditional production and operation methods within this group is relatively small. At the same time, such enterprises focus on the practical application of technology and fully sink the limited digital technology into the business process. To improve the efficiency of digital technology utilization, they continue to accelerate the integration of digital technology into business activities and promote the transformation of business practices. Thus, it promotes DT to empower enterprises and improve their ESG performance. The enterprises that fit this model are shown in Fig. 8.

**Figure 9:**
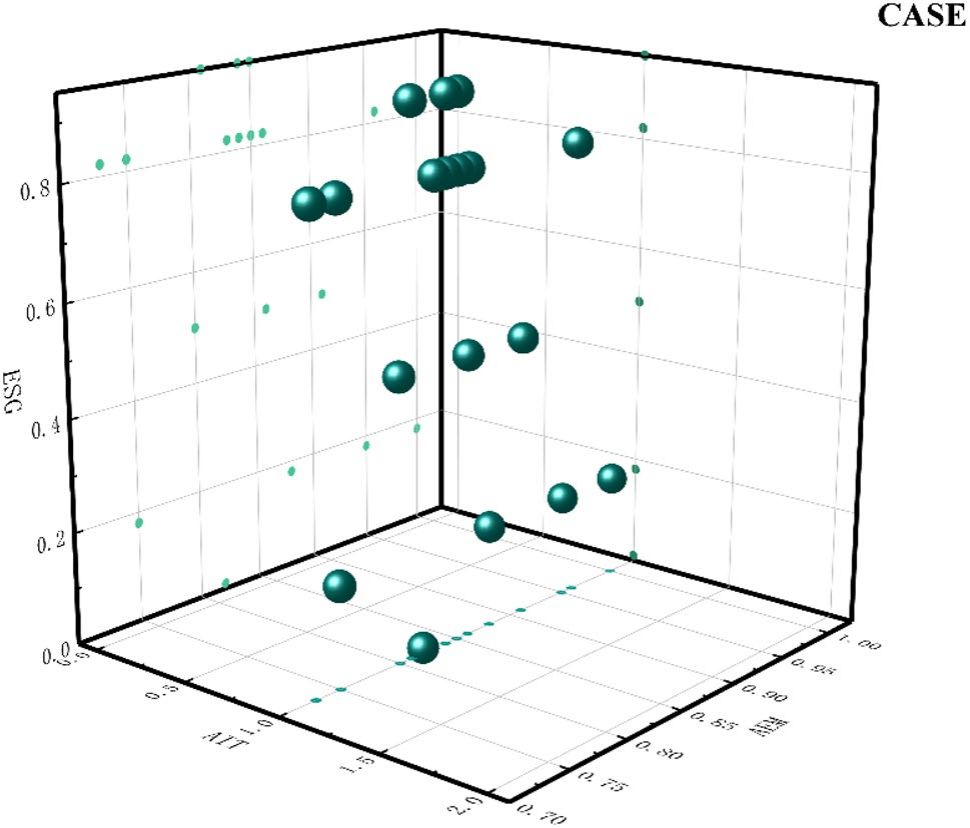
Path 6 case diagram.

Path 6: AIT*EM* ~ TPA* ~ BT is that enterprises with insufficient digital technology practice and low BT rely on AIT and high EM to drive ESG performance. These companies rely on digital intelligence and high EM to drive ESG. The application of AI and automation technologies has revolutionized the economic activities and decision-making behavior of firms^68,69^. This category of companies relies on the decision-making advantages of AI to compensate for the lack of development of digital application scenarios. Among the internal conditions of DT, the main reliance is on digital intelligence to drive sustainable development. Digital intelligence creates a database of ESG development status to help companies analyze and manage data dynamics. It helps companies assess their sustainability progress, reduce the negative impact of their daily operations on the environment, and build a more responsible and resilient supply chain to meet their ESG development goals. At the same time, this category of companies has high EM practices. It indicates that enterprises have high-performance manipulation in the process of using digital intelligence to enable sustainable development. The rest of the digital development is generally absent. The enterprises that fit this model are shown in Fig. 9.

### Discussion

First, DT can significantly reduce enterprise ESG performance, with an impact coefficient of − 0.013, which is significant at the 5% level. Corporate sustainability is distinguished from traditional performance evaluation. It emphasizes the positive impact of business operations on the surrounding environment and includes the business owner as one of the affected objects^70^. In the process of DT, dynamic capability exploration intensifies the instability of the organizational system, and numerous hidden costs further reduce the profit space of enterprises^71^. DT is a long-term process, and there is a time lag between the enterprise's organizational status and the digital technology architecture. It is necessary to gradually carry out DT according to the development situation of the enterprise, the industry situation, and the enterprise planning. The introduction and skilled application of digital technology also require increased management costs and substantial investment in innovation. Innovation activities require long-term factor input, which leads to a decline in ESG investment^72^. In fact, as a business organization, "profit-seeking" is its most prominent feature. In the process of business activities, enterprises take the maximization of interests as the basic goal. When faced with the impact of DT, enterprises tend to reduce their attention to other stakeholders. The focus on environmental responsibility, social responsibility, and internal governance will be reduced, and more resources will flow to business activities, thus significantly reducing ESG performance.

Second, DT has a significant inhibitory effect on AEM, with an impact coefficient of − 0.196, which is significant at the 10% level. The effect on REM is relatively small and has no statistical significance. DT can inhibit EM from both internal and external aspects. Within enterprises, DT can accurately depict market demand and customer feedback, thus enhancing the effectiveness of production. It reduces the motivation and inclination of management to implement EM^38^. In the aspect of enterprise external supervision, DT converts massive business information into data. It improves the information asymmetry of enterprises and reduces the cost of external supervision, which restrains the EM activities of the management. The effect on REM is not significant, which proves that REM has a certain concealment and is more difficult to detect than AEM, so the DT has a more obvious inhibitory effect on AEM^73^. Therefore, the rationality of accrued earnings management as a suppression variable is stronger.

Third, under the suppression model, AEM has a significant inhibitory effect on ESG performance, with a coefficient of − 0.109, which is significant at the 5% level. The adverse effect of DT on ESG decreased from − 0.013 to − 0.009, with no statistical significance. The indirect effect results are contrary to the direct effect results, reflecting that the AEM has a suppression effect. Before digital technology truly enables enterprises to operate, DT will have a certain impact on the traditional mode of operation. The integration degree with business activities is insufficient, which makes it difficult to take into account ESG performance. However, with the continuous collection and collation of digital technology, the problem of information asymmetry can be improved. Thus, it can limit the change of accounting income information and restrain the management of accrued earnings. The improvement of information transparency can increase the sense of responsibility of enterprises and promote the establishment of an external image^74^. The transparency of information also drives the transparency of operations, and the requirements for internal governance increase accordingly. Therefore, the impact of DT on ESG performance decreases.

Fourth, we find six configuration paths of DT and EM driving ESG performance. First, high technology practice—low-performance manipulation. Technology practice reduces the impact of DT on enterprise management, increases new business scenarios, and promotes the integration of digital technology and enterprise management^75^. Most of these companies lack BT, while other underlying technologies are relatively well-developed. BT has immutability issues, information leakage risks, and regulatory issues that hinder the development of the technology's application. Scholars have proposed a blockchain-based security digital framework: Block-DEF^76^. The framework has a loosely coupled structure to distinguish evidence from evidence information. Only evidence information is stored in the blockchain, and evidence is stored on a trusted storage platform. It can ensure the integrity and validity of evidence while striking a good balance between privacy and traceability. In the process of future DT, blockchain technology needs to be further developed and expanded to make up for existing application deficiencies. Second, digital intelligence – low-performance manipulation. Some enterprises have relatively weak digital technology infrastructure and focus on digital intelligence to cultivate sustainable development advantages based on internal differential data. At the same time, such enterprises continue to enhance financial credibility, reduce information asymmetry, and establish a good corporate image. Third, digital intelligence—digital resources. In the later stage of DT, enterprises can organize massive data with BDT and extract core digital information in a short time^77^. Processing has become a digital resource that can improve the sustainable development of enterprises, and scientific decision-making using digital intelligence has effectively improved the level of enterprise ESG. Then processing them into digital resources that can improve the sustainable development of enterprises, and scientific decision-making using digital intelligence effectively improves the level of enterprise ESG. Fourth, digital resources—inadequate digital infrastructure. Some enterprises generally lack infrastructure construction and mainly rely on digital resources as the core conditions, which further reflects the digital resource advantages of BDT. Fifth, high technology practice—bottom technology deficiency. When the underlying digital technologies are weak, such enterprises pay attention to the practical application of digital technologies, actively utilize limited digital technologies in their business operations, and promote the level of ESG by expanding green business scenarios. Sixth, digital intelligence—high-performance manipulation. This kind of enterprise has a single digital technology and focuses on the decision analysis ability of artificial intelligence. This category focuses on the development of digital intelligence technologies in the process of DT. High-performance manipulation is also adopted to guide external investment orientation. Investments in blockchain and digital technology use are decreasing and converging resources to improve ESG.

## Conclusions

The main conclusions of this paper are as follows: First, DT can significantly reduce ESG performance. This reflects that there are certain shortcomings in the DT of enterprises at the current stage. There is a matching lag in the internal organizational resources of enterprises, and the entry of digital technology will have some impact on the traditional operation of enterprises. The high uncertainty of DT adds some hidden costs to enterprises. Second, there is a suppression effect of AEM in the transmission mechanism of DT on ESG. It is verified that DT can reduce information asymmetry and inhibit EM, thus reducing the impact on ESG performance. Finally, the high valuation of ESG is the result of a combination of factors. The suppression effect of EM is further validated by configuration analysis. The absence of AEM usually serves as a core condition for high-valuation configurations. Meanwhile, digital intelligence, digital resources, and digital technology practices can drive corporate ESG more. Based on the theoretical analysis and empirical findings, this paper proposes the following policy recommendations.

(1) First, enterprises should increase strategic flexibility in the process of DT to reduce the impact effect of digital technology. Enterprises should reduce their path dependence on traditional technologies and cultivate dynamic core competencies through DT. At the same time, enterprises should enhance cooperation to jointly develop digital underlying technologies and share resources and knowledge to cope with different challenges and changes. To improve the degree of digital resource transformation, companies can combine operational data and user needs to monitor market dynamics and demand in real-time. Companies can also establish intelligent systems to analyze data and generate predictive models to improve the conversion rate of digital resources, thereby increasing competitiveness and market share. For competence flexibility, companies need to develop a clear digital plan to develop employees' digital competencies such as the application of digital technology, data analysis, and innovative thinking. In this way, employees can combine their existing work experience and quickly adapt to the work at hand. (2) External supervision should rely on the DT mechanism to restrain earnings manipulation. Regulatory authorities should actively take advantage of digital technology, carry out new regulatory projects, and make extensive and effective use of digital technologies such as big data and artificial intelligence. A unified supervisory data platform needs to be established to facilitate data sharing among regulators. Based on digital technology, enterprise data can be collected, abnormal activities can be recorded and analyzed, EM probability can be assessed, and earnings activity probability classification can be achieved. At the same time, high-risk enterprises should be supervised selectively to improve supervision efficiency. In the future, digital tracking technology should be further used for high-frequency monitoring and outlier tracking, and supervision subject and content should be expanded to achieve effective supervision. In addition, social platforms and news media should play a role in strengthening public opinion supervision, promoting corporate introspection, and reducing performance manipulation. (3) Enterprises should pay attention to the multi-dimensional expansion of digital technology and promote the coordinated development of underlying technology and technology applications. Companies should consider the match between digital technology and their development model. It is necessary to combine a variety of digital technologies reasonably, strengthen digital thinking, and improve the efficiency of resource allocation. To innovate the business model and strategy, give full play to the advantages of DT, and break through the development bottleneck. Enterprises should give priority to developing highly matched digital technologies, and promote ESG performance with practical applications.

### Supplementary Information

Supplementary Information 1.

Supplementary Information 2.

## Data Availability

The datasets generated during and/or analyzed during the current study are available from the corresponding author upon reasonable request.
