# Supplementary material for: The impact of digital transformation and earnings management on ESG performance: evidence from Chinese listed enterprises
Source: Sci Rep. 2024 Jan 8;14:783. doi: 10.1038/s41598-023-48636-x (PMC10774430; doi:10.1038/s41598-023-48636-x)
Supplement: Supplementary file 1 — Supplementary Information 1. [file 41598_2023_48636_MOESM1_ESM.docx]

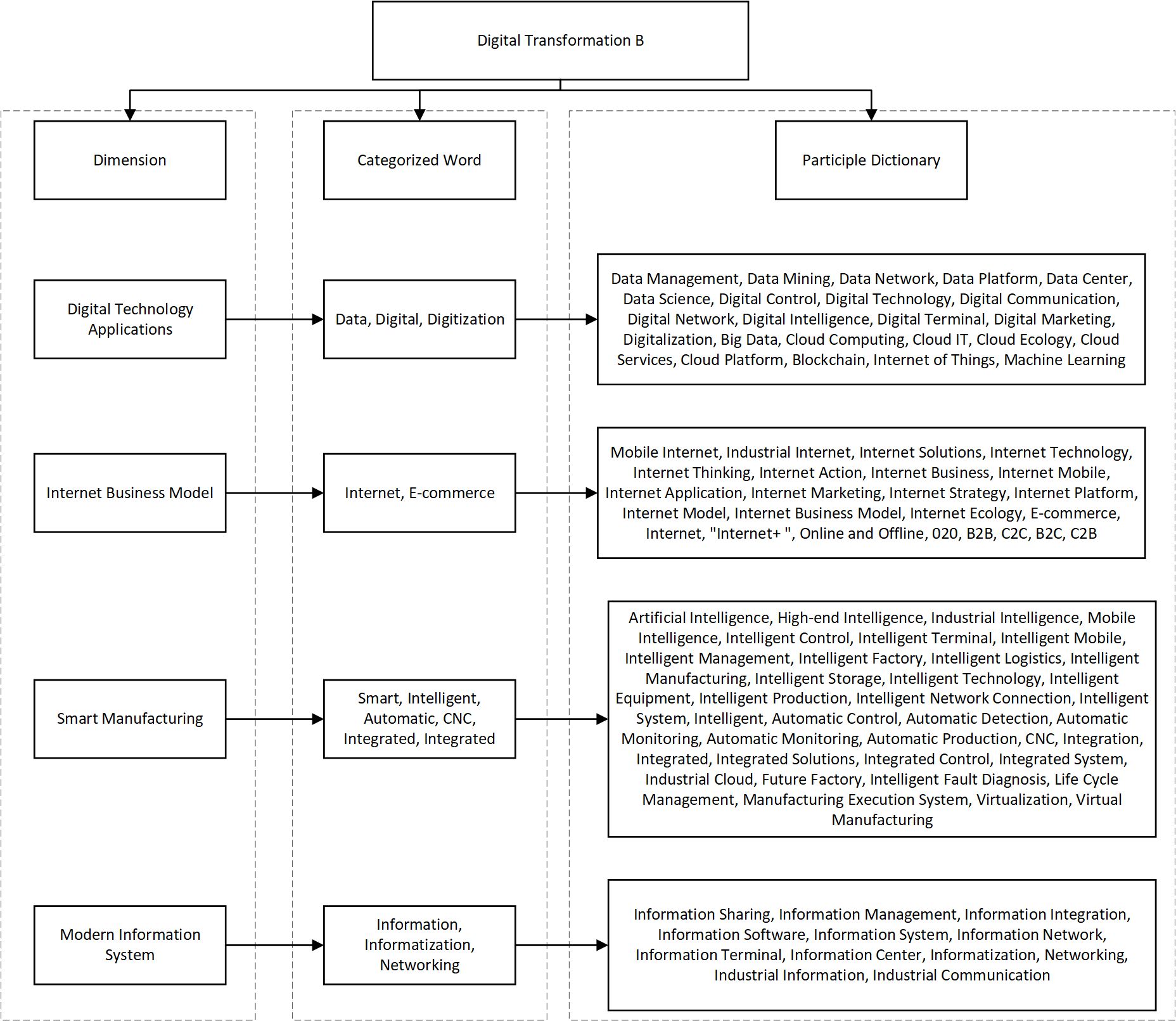


Appendix Figure S1 Independent Variable Reconfiguration

Appendix Table S1 Truth Table

| AIT | BDT | CCT | BT | TPA | AEM | Number | ESG | raw  consist. | PRI  consist. | SYM  consist |
| --- | --- | --- | --- | --- | --- | --- | --- | --- | --- | --- |
| 0 | 1 | 0 | 0 | 1 | 1 | 9 | 1 | 0.858 | 0.5097 | 0.5165 |
| 0 | 0 | 0 | 0 | 1 | 1 | 31 | 1 | 0.817 | 0.5147 | 0.5270 |
| 0 | 1 | 0 | 0 | 0 | 1 | 10 | 1 | 0.8172 | 0.4812 | 0.4823 |
| 1 | 1 | 0 | 0 | 0 | 1 | 4 | 1 | 0.8090 | 0.5227 | 0.5530 |
| 1 | 1 | 0 | 0 | 1 | 1 | 9 | 1 | 0.8050 | 0.5549 | 0.5618 |
| 1 | 0 | 0 | 0 | 0 | 1 | 7 | 1 | 0.7722 | 0.5206 | 0.5299 |
| 1 | 1 | 1 | 0 | 0 | 1 | 13 | 1 | 0.7711 | 0.5400 | 0.544 |
| 1 | 0 | 1 | 0 | 0 | 1 | 5 | 1 | 0.765 | 0.5058 | 0.5158 |
| 1 | 1 | 0 | 0 | 0 | 0 | 6 | 1 | 0.8484 | 0.6078 | 0.6235 |
| 0 | 1 | 0 | 0 | 1 | 0 | 8 | 1 | 0.8477 | 0.5025 | 0.5068 |
| 1 | 1 | 1 | 0 | 0 | 0 | 9 | 1 | 0.8032 | 0.5861 | 0.5961 |
| 0 | 0 | 0 | 0 | 1 | 0 | 25 | 1 | 0.7985 | 0.4740 | 0.4878 |
| 1 | 0 | 0 | 0 | 1 | 0 | 5 | 1 | 0.7916 | 0.5033 | 0.5121 |
| 1 | 1 | 0 | 0 | 1 | 0 | 4 | 1 | 0.7901 | 0.4872 | 0.4990 |
| 0 | 1 | 0 | 0 | 0 | 0 | 11 | 1 | 0.7868 | 0.4497 | 0.4531 |
| 1 | 1 | 1 | 0 | 1 | 0 | 43 | 1 | 0.7803 | 0.6174 | 0.6395 |
| 0 | 1 | 1 | 0 | 1 | 0 | 10 | 1 | 0.7672 | 0.5618 | 0.5653 |
| 1 | 0 | 1 | 0 | 1 | 0 | 10 | 1 | 0.7657 | 0.5255 | 0.5262 |
| 0 | 0 | 1 | 0 | 1 | 0 | 6 | 1 | 0.7573 | 0.5004 | 0.5151 |
| 1 | 0 | 0 | 0 | 0 | 0 | 5 | 1 | 0.7550 | 0.4892 | 0.5025 |
| 1 | 1 | 1 | 1 | 1 | 0 | 29 | 1 | 0.7534 | 0.5516 | 0.6074 |
