# Supplementary material for: The impact of digital transformation and earnings management on ESG performance: evidence from Chinese listed enterprises
Source: Sci Rep. 2024 Jan 8;14:783. doi: 10.1038/s41598-023-48636-x (PMC10774430; doi:10.1038/s41598-023-48636-x)
Supplement: Supplementary file 2 — Supplementary Information 2. [file 41598_2023_48636_MOESM2_ESM.docx]

# DT

import xlwt

import os

def matchKeyWords(txt_folder, keyWords):

files = os.listdir(txt_folder)

words_num = []

for file in files:

word_freq = {}

if os.path.splitext(file)[-1] == ".txt":

txt_path = os.path.join(txt_folder, file)

with open(txt_path, "r", encoding='utf-8', errors='ignore')as fp:

text = fp.readlines()

for word in keyWords:

num = 0

for line in text:

num += line.count(word)

word_freq[word] = num

stock_code = file.split("_")[1:6]

stock_name = file.split("_")[6:10]

year = file.split("_")[10:]

words_num.append((word_freq, stock_code, stock_name, year))

book = xlwt.Workbook(encoding='utf-8', style_compression=0)

sheet = book.add_sheet(' Annual keyword frequency statistics', cell_overwrite_ok=True)

sheet.write(0, 0, '')

sheet.write(0, 1, '')

sheet.write(0, 2, '')

sheet.write(0, 3, '')

sheet.write(0, 3, '')

for index, one in enumerate(words_num):

word_f = one[0]

stock_code = one[1]

stock_name = one[2]

year = one[3]

for ind, word in enumerate(keyWords):

sheet.write(index + 1, ind + 3, str(word_f[word]))

sheet.write(index + 1, 0, stock_code)

sheet.write(index + 1, 1, stock_name)

sheet.write(index + 1, 2, year)

book.save(r'E:\ Annual keyword frequency statistics.xls')

import excel Raw word frequency data.xlsx , sheet("Sheet1") firstrow

gen AI =rowtotal(AI, Business Intelligence, Image Understanding, Investment Decision Assistance Systems, Intelligent Data Analysis, Intelligent Robots, Machine Learning, Deep Learning, Semantic Search, Biometrics, Facial recognition, Speech Recognition, Authentication, Automatic Driving, Natural Language Processing)

egen BD =rowtotal(Big Data, Data Mining, Text Mining, Data visualization, Heterogeneous data, Credit Investigation, Augmented Reality, Mixed reality, Virtual Reality)

egen CC=rowtotal(Cloud computing, Flow computing, Graph Computing, Memory Computing, Multi-party Security Computing, Brain-like Computing, Green Computing, Cognitive Computing, Fusion Architecture, Giga-level concurrency, EB-level Storage, Iot, Information Physical System)

egen Block =rowtotal(Blockchain, Digital currency, Distributed Computing, Differential Privacy Technolog,y Smart Financial Contract)

egen App=rowtotal(Mobile Internet Industrial Internet, Mobile Internet, Medical Internet, E-commerce mobile payment, Third party payment NFC payment Smart Energy B2B, B2C, C2B, C2C, O2O, Network connected, Smart Wearable, Smart Agriculture, Smart Transportation, Smart t medical, Smart customer service, Smart home, Smart Investment, Smart Travel, Smart Environmental Protection, Smart Grid, Smart Marketing, Digital Marketing, Unmanned Retail, Internet Finance, Digital Finance, Fintech Quantitative Finance, Open Bank)

egen sum =rowtotal(Artificial Intelligence Technology, Big Data Technology, Cloud Computing technology, Blockchain technology, Digital technology Application)

gen DT=ln(sum+1)

gen AIT=ln(1+AI)

gen BT=ln(1+Block)

gen CCT=ln(1+CC)

gen BDT=ln(1+BD)

gen ADT=ln(1+App)

# AEM

use "",clear

replace induscode=substr(induscode,1,2) if substr(induscode,1,1)=="C"

replace induscode =substr(induscode,1,1) if substr(induscode,1,1)!="C"

drop if induscode =="J"

egen ind=group(induscode)

gen ST=strmatch(stock, "*ST*")

drop if ST==1

destring code,replace

xtset code year

gen lag_T=l.T

gen lag_REV =l.REV

gen lag_REC =l.REC

gen lag_TA =l.TA

bys code year:gen B1=1/lag_T

bys code year:gen B2=(REV-lag_REV)/lag_T

bys code year:gen B3=PPE/lag_T

bys code year:gen B4=((REV-lag_REV)-(REC-lag_REC))/lag_T

gen y=TA/lag_T

xtset code year

sort code year

egen mis=rowmiss(B1 B3 B4 y ind)

drop if mis

xtset code year

sort code year

winsor2 y B1 B3 B4,replace cuts(1 99)

gen e=.

egen indnum=group(ind)

qui: sum indnum

local indmax=r(max)

forvalues i=1/`indmax'{

forvalues j=2011/2021 {

capture reg y B1 B3 B4 if indnum==`i'&year==`j'

if !_rc {

predict double rs if e(sample), residuals

replace e=rs if e(sample)

drop rs

}

}

}

rename e DA2

drop if DA2==.

gen absDA2=abs(DA2)

sort code year

save ""

# REM

use "",clear

replace induscode=substr(induscode,1,2) if substr(induscode,1,1)=="C"

replace induscode=substr(induscode,1,1) if substr(induscode,1,1)!="C"

drop if induscode=="J"

egen ind=group(induscode)

gen ST=strmatch(stock, "*ST*")

drop if ST==1

rename Inventory INV

rename Total asset T

rename Revenue REV

rename Operating cost COGS

rename Sale expense SC

rename Management fee MC

rename Net cash flows from operating activities CFO

xtset code year

gen lag_T=l.T

gen lag_REV =l.REV

gen lag2_REV =l2.REV

gen lag_INV =l.INV

gen DS=REV-lag_REV

gen DS_2=lag_REV-lag2_REV

bys code year:gen CFO_A=CFO/lag_T

bys code year:gen B1=1/lag_T

bys code year:gen B2=REV/lag_T

bys code year:gen B3=DS/lag_T

bys code year:gen PROD_A=(COGS+(INV-lag_INV))/lag_T

bys code year:gen B4=DS_2/lag_T

bys code year:gen DISP_T=(SC+MC)/lag_T

bys code year:gen B5=lag_REV/lag_T

xtset code year

sort code year

save ""

use "",clear

keep code year CFO_A B1 B2 B3 ind

egen mis=rowmiss(CFO_A B1 B2 B3 ind)

drop if mis

xtset code year

sort code year

gen e=.

egen indnum=group(ind)

qui: sum indnum

local indmax=r(max)

forvalues i=1/`indmax'{

forvalues j=2011/2022 {

capture reg CFO_A B1 B2 B3 if indnum==`i'&year==`j'

if !_rc {

predict double rs if e(sample), residuals

replace e=rs if e(sample)

drop rs

}

}

}

rename e ABCFO

label var ABCFO " Abnormal cash flow from operating activities"

keep code year ind ABCFO

sort code year

save ""

use "",clear

keep code year PROD_A B1 B2 B3 B4 ind

egen mis=rowmiss(PROD_A B1 B2 B3 B4 ind)

drop if mis

xtset code year

sort code year

gen e=.

egen indnum=group(ind)

qui: sum indnum

local indmax=r(max)

forvalues i=1/`indmax'{

forvalues j=2011/2022 {

capture reg PROD_A B1 B2 B3 B4 if indnum==`i'&year==`j'

if !_rc {

predict double rs if e(sample), residuals

replace e=rs if e(sample)

drop rs

}

}

}

rename e ABPROD

label var ABPROD " Abnormal product cost "

keep code year ind ABPROD

sort code year

save ""

keep code year DISP_A B1 B5 ind

egen mis=rowmiss(DISP_A B1 B5 ind)

drop if mis

xtset code year

sort code year

gen e=.

egen indnum=group(ind)

qui: sum indnum

local indmax=r(max)

forvalues i=1/`indmax'{

forvalues j=2011/2022 {

capture reg DISP_A B1 B5 if indnum==`i'&year==`j'

if !_rc {

predict double rs if e(sample), residuals

replace e=rs if e(sample)

drop rs

}

}

}

rename e ABDISP

label var ABDISP " Abnormal discretionary expenses "

keep code year ind ABDISP

sort code year

save ""

use "",clear

merge 1:m code year using ""

drop _merge

merge 1:m code year using ""

drop _merge

xtset code year

sort code year

gen REM=ABPROD-ABCFO-ABDISP

sort code year

save ""
